# Far-Red Fluorescent Murine Glioma Model for Accurate Assessment of Brain Tumor Progression

**DOI:** 10.3390/cancers14153822

**Published:** 2022-08-06

**Authors:** Tatiana A. Mishchenko, Irina V. Balalaeva, Maria O. Klimenko, Anna A. Brilkina, Nina N. Peskova, Evgenii L. Guryev, Dmitri V. Krysko, Maria V. Vedunova

**Affiliations:** 1 Department of Neurotechnology, Institute of Biology and Biomedicine, Lobachevsky State University of Nizhny Novgorod, 23 Gagarin Ave., 603022 Nizhny Novgorod, Russia; 2 Department of Basic and Medical Genetics, Institute of Biology and Biomedicine, Lobachevsky State University of Nizhny Novgorod, 23 Gagarin Ave., 603022 Nizhny Novgorod, Russia; 3 Department of Biophysics, Institute of Biology and Biomedicine, Lobachevsky State University of Nizhny Novgorod, 23 Gagarin Ave., 603022 Nizhny Novgorod, Russia; 4 Cell Death Investigation and Therapy Laboratory (CDIT), Anatomy and Embryology Unit, Department of Human Structure and Repair, Ghent University, C. Heymanslaan 10, Building B3, 4th Floor, 9000 Ghent, Belgium; 5 Cancer Research Institute Ghent, 9000 Ghent, Belgium

**Keywords:** glioma, GL261 cells, fluorescent, diagnostic, tumor growth, orthotopic model, brain cancer

## Abstract

**Simple Summary:**

The creation of stable fluorescent glioma cell lines opens prospects for the detailed characterization of the molecular mechanisms of glioma origin and pathogenesis, and the development of breakthrough therapeutic approaches to achieve maximum glioma destruction and minimize the risk of future metastases. Herein, we generated and characterized a novel fluorescent glioma GL261-kat cell line stably expressing a far-red fluorescent protein, TurboFP635 (Katushka). Using the orthotopic mouse glioma model and epi-fluorescence imaging, we demonstrate the detection of fluorescent glioma GL261-kat cells in mice, and observe an increase in the fluorescence signal during glioma progression, which is accompanied by a gradual development of neurological deficit and behavioral alterations in mice. We show that GL261-kat cells can be a useful tool for studying glioma biology, because they can accurately and non-invasively monitor the characteristics of glioma growth in brain tissue in orthotopic mouse models.

**Abstract:**

Glioma is the most common brain tumor, for which no significant improvement in life expectancy and quality of life is yet possible. The creation of stable fluorescent glioma cell lines is a promising tool for in-depth studies of the molecular mechanisms of glioma initialization and pathogenesis, as well as for the development of new anti-cancer strategies. Herein, a new fluorescent glioma GL261-kat cell line stably expressing a far-red fluorescent protein (TurboFP635; Katushka) was generated and characterized, and then validated in a mouse orthotopic glioma model. By using epi-fluorescence imaging, we detect the fluorescent glioma GL261-kat cells in mice starting from day 14 after the inoculation of glioma cells, and the fluorescence signal intensity increases as the glioma progresses. Tumor growth is confirmed by magnetic resonance imaging and histology. A gradual development of neurological deficit and behavioral alterations in mice is observed during glioma progression. In conclusion, our results demonstrate the significance and feasibility of using the novel glioma GL261-kat cell line as a model of glioma biology, which can be used to study the initialization of glioma and monitor its growth by lifetime non-invasive tracking of glioma cells, with the prospect of monitoring the response to anti-cancer therapy.

## 1. Introduction

Despite significant progress in neurosurgery, and the ongoing improvements of combined therapeutic approaches to glioma treatment, the survival rate remains very low and prognosis poor. In 2021, the World Health Organization (WHO) presented an updated Classification of Tumors of the Central Nervous System, which categorized five glioma subtypes according to their morphological, molecular, and genetic profiles [1,2]. Gliomas constitute about 77% of all neuroepithelial tumors, and are characterized by high histological heterogeneity, rapid invasive growth, high proliferation rate, and intensive angiogenesis [3,4]. The five year survival rate for patients with glioma ranges from 66% among young patients (from infancy to 19 years) to 5% in the elderly (≥75 years). For glioblastoma multiforme (WHO grade IV), the survival rates are 13% (20–44 years) and 1% (55–64 years), respectively [5,6,7,8]. To lengthen the life expectancy and enhance the quality of life of patients with gliomas, there is an urgent need to characterize glioma development in detail, and to develop breakthrough therapeutic approaches to achieve maximum glioma destruction and minimize the risk of future metastases.

Murine glioma models are important for studying glioma’s origin and pathogenesis [9,10,11], because they offer the possibility of exploring in detail different aspects of glioma biology, including morphological, genetic, biochemical, and functional alterations of the brain tissue during tumorigenesis, as well as the identification of specific diagnostic and prognostic glioma biomarkers, development of effective new anti-cancer strategies, and development of next-generation drugs.

The creation of stable tumor cell lines labeled with genetically encoded fluorescent proteins is a promising tool in experimental oncology. Fluorescent tumor models have several advantages compared to non-fluorescent tumor models. For instance, fluorescently labeled tumor cell lines can be used for tracking the precise molecular mechanisms of tumor growth, determining tumor cell death rate, and monitoring the activation of signaling pathways in response to anti-cancer therapy [12,13,14,15,16]. Moreover, the fluorescent proteins can function as additional tumor antigens that help in the study of anti-tumor immune responses [17,18], or can be used for in vivo fluorescent imaging to monitor tumor growth patterns, metastatic processes, and tumor regression. In addition, fluorescent tumor models enable the assessment of the peculiarities of tumor invasion, and reveal the intensity of blood supply to the tumor [19,20,21,22,23,24]. Therefore, the use of in vivo tumor models based on fluorescently labeled cell lines is especially relevant to the study of tumors that are highly invasive and have no clear neoplasm boundaries, such as gliomas: they make it possible to track single tumor cells remaining after surgery and anti-cancer therapy.

Among the various fluorescent proteins with different spectral characteristics, particular preference is given to the creation of fluorescently labeled cell lines to red and far-red proteins (625–900 nm) [25]. These fluorescent proteins have greater light penetration, entering the therapeutic window of biological tissue transparency, and can be visualized at a depth of up to one centimeter [14,26,27,28]. The pronounced fluorescence emission maxima in the visible and infrared spectral region minimize the influence of tissue autofluorescence and scattered excitation radiation on the registration of the fluorescence signal of interest.

Herein, we present a novel fluorescent glioma GL261-kat cell line stably expressing a far-red fluorescent protein, TurboFP635, also known as Katushka, which has an emission maximum at 635 nm. We describe a step-by-step procedure for obtaining and characterizing monoclonal GL261-kat glioma cells and their validation in a mouse orthotopic glioma model. In this model, we demonstrate the accurate monitoring of glioma growth in the brain using epi-fluorescence imaging, and assess the impact of the developing tumors on the severity of neurological deficit, as well as on the cognitive and learning abilities of mice. A complex analysis of the morphological changes of brain tissue is also presented.

## 2. Material and Methods

### 2.1. Cell Culture and Transfection

The murine glioma cells, GL261, were used as a parental cell line. Murine glioma GL261 cells were kindly provided by Prof. Dr. P. Agostinis, Laboratory of Cell Death Research and Therapy, Department of Cellular and Molecular Medicine, KU Leuven (Leuven, Belgium). The cells were cultured in DMEM medium containing 4.5 g/L glucose (PanEco, Moscow, Russia) and supplemented with 2 mM glutamine (PanEco, Russia), 100 μM sodium pyruvate (Thermo Fisher, Waltham, MA, USA), and 10% fetal bovine serum (Thermo Fisher, USA). They were cultured at 37 °C in a humidified atmosphere containing 5% CO_2_ in a Binder C150 incubator (BINDER GmbH, Tuttlingen, Germany). At the end of the exponential growth period, the cells were removed with a trypsin–versene solution (1:3) (PanEco, Russia) and reseeded at a multiplicity of seeding of 1:10, and an approximate cell density of 1.0 × 10^5^ cells/mL.

The parental cell line GL261 was transfected with the plasmid vector pTurboFP635-N (Evrogen, Moscow, Russia), in which the gene for TurboFP635 fluorescent protein is under the control of the cytomegalovirus promoter, which enables protein expression in mammalian cells. Lipofection was carried out using Lipofectamin 3000 reagent (Invitrogen, Waltham, MA, USA), followed by primary selection of cells in medium supplemented with increasing concentrations up to 2 mg/mL of an aminoglycoside antibiotic, G418 (Invitrogen, USA). The transfected cells were then sorted using a FACS Aria III cell sorter (BD Biosciences, Franklin Lakes, NJ, USA). Cells with the strongest TurboFP635 protein fluorescence (λex 561 nm and λem 600–630 nm) were sorted, expanded in cell culture, and sorted again to obtain the brightest fluorescent cell subset. Three sort/expand cycles were used. At the last stage, we applied a ‘single cell sorting’ mode, which allows for the collection of one cell per well in a 96-well culture plate. Following cultivation, the individual monoclonal colonies were obtained. The clone that maintained the morphology and growth rate of the parental line and had the strongest TurboFP635 fluorescence was selected and established as the monoclonal GL261-kat cell line. Fluorescence detection of TurboFP635 in transfectant glioma GL261 cells was performed using an Axio Observer Z1 LSM-710 DUO NLO laser scanning microscope (Carl Zeiss, Jena, Germany) with an LD C-Apochromat water immersion objective lens with 40× magnification and numerical aperture of 1.1. For image acquisition, the fluorescence was excited at 594 nm and the signal was recorded in the 603–720 spectral range.

### 2.2. Mice and Ethics Statement

In vivo studies were performed on female BALB/c mice (5–7 weeks old). The use of allogenic mice contributed to a decrease in the intensity of tumor growth in the orthotopic glioma model [29,30] and, thus, provided the opportunity for long-term monitoring of the intensity of the fluorescent signal of inoculated glioma cells in the brains of mice. The animals were housed in a conventional vivarium of Lobachevsky State University of Nizhny Novgorod. All experimental procedures were approved by the Bioethics Committee of Lobachevsky University (ethic code No57 from 15 November 2021), and carried out in accordance with Act 708n (23 082010) of the Russian Federation National Ministry of Public Health, which states the rules of laboratory practice for the care and use of laboratory animals, and Council Directive 2010/63 EU of the European Parliament (22 September 2010) on the protection of animals used for scientific purposes.

### 2.3. Surgical Procedures and Orthotopic Transplantation of Glioma GL261-Kat Cells In Vivo

The mice were anesthetized by inhalation of Isoflurane (Laboratorios Karizoo, Barcelona, Spain) at a concentration of 1.5–2.0% in a gas-air mixture. In the absence of proprioceptive reflexes, the top of the animal’s head was cleared of fur and fixed in a stereotactic frame (World Precision Instruments, Sarasota, FL, USA). After soft tissue incision, a trepanation window in the right hemisphere was formed using a fine drill according to the stereotactic coordinates (2 mm lateral and 2 mm posterior to the bregma). The mice were injected with 2.5 × 10^4^ (GL25 group) or 5 × 10^4^ (GL50 group) glioma GL261-kat cells suspended in 2 μL of phosphate-buffered saline (PBS, Invitrogen, Waltham, MA, USA). Injection was carried out intracranially, 2.5 ± 0.5 mm below the dura mater. The control mice were injected with 2 µL of PBS. For intravital studies of the dynamics of fluorescent signal propagation of glioma GL261-Kat cells, the trepanation window was closed with a sterile cover glass fixed to the skull with a dental composite (DentLight-flow, Vladmiva, Belgorod, Russia). The material was polymerized with a LUX V lamp (Woodpecker/DTE, Guangxi, China). The adjacent tissues were tightly sutured with surgical thread (0.2 mm) and treated with an antiseptic solution. After the procedure, the animals were allowed to recover from anesthesia before being returned to their cages with postoperative care and ad libitum access to food and water.

### 2.4. Epi-Fluorescence Imaging In Vivo

**Figure 1:**
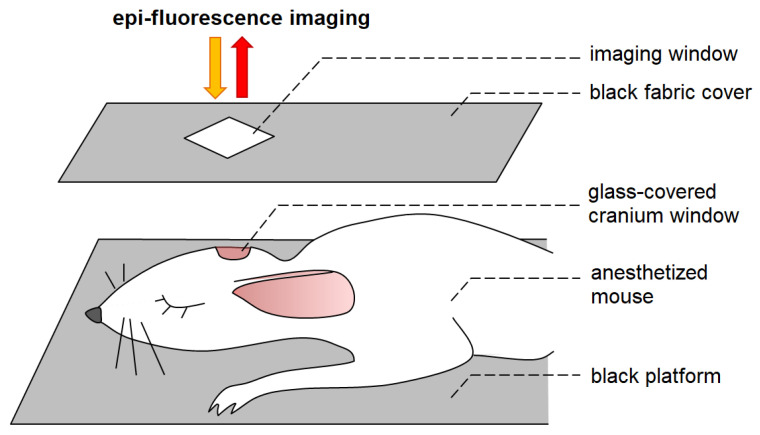
Representation of the animal position during the epi-fluorescence imaging.

The features of tumor progression after inoculation of glioma GL261-kat cells were analyzed by epi-fluorescence imaging. Before the procedure, the mice underwent general anesthesia (intraperitoneal injection of 70 mg/kg Zoletil^®^100 (Virbac Sante Animale, Carros, France) followed by intramuscular injection of 0.01 mg/kg Xilanit (Nita-Farm, Saratov, Russia), and fixed on a black platform. They were covered with black fabric to minimize the effects of scattered light and autofluorescence on the signal of interest (Figure 1). A glass skull cover with a square hole for imaging the region of interest was placed on the head of the mice.

A whole-body imaging system (Deep Vision System, DVS-03, Federal Scientific Research Centre “Crystallography and Photonics” of the RAS, Moscow, Russia) was used with TurboFP635 excitation at 590 nm and detection of fluorescence in the region of 625–675 nm.

### 2.5. Magnetic Resonance Imaging

Magnetic resonance imaging (MRI) was performed by using a high-field magnetic resonance tomograph (Agilent Technologies DD2–400 9.4 T; 400 MHz; Cheadle, UK) with a volume coil M2M (H1). The anesthetized mice were kept in a fixed position inside the magnet tunnel for 40 min. The MR images were obtained and analyzed using VnmrJ program. T1- and T2-tomograms of layer-by-layer frontal brain sections weighted by proton density were performed using the multi gradient echo multi slice (MGEMS) pulse sequence with the following parameters: TR = 1000 ms, TE = 1.49 ms, 6 echoes, FOV 20 × 20 mm, matrix 256 × 256, slice thickness 1 mm, 15 slices, 17 min and 4 s scanning time.

### 2.6. Neurological Status Determination

The functional state of the CNS was assessed by a severity scale of neurological deficit with modifications for mice [31,32,33]. The scale includes 10 tests of motor activity, coordination of movements, reflexes, muscle tone, and ptosis and exophthalmos; each of these is scored 2 points for no reaction, 1 for some disturbances, and 0 for good/normal reaction. The values were summarized and interpreted according to the following grade: 10–20 points: severe CNS damage; 6–9 points: moderate CNS damage; and 1–5 points: light CNS damage.

### 2.7. Open Field Test

The general locomotor and orienting-exploratory activity of the mice were tested in the open field box (LE800S; Panlab Harvard Apparatus, Barcelona, Spain). The behavioral reactions were recorded for 5 min using a Sony SSC-G118 camera (Tokyo, Japan). The following parameters were assessed: general motor activity (number of the squares crossed in the center and periphery of the arena), vertical motor activity (number of upright postures), and emotional state (number of grooming acts, defecation and urination, time spent in the center of the arena).

### 2.8. Passive Avoidance Test

The learning ability of the mice was assessed using a chamber (60 cm × 20 cm × 25 cm) with an electrified lattice floor divided into darkened and lighted sections (Shuttle Box LE918; Panlab Harvard Apparatus, Spain). The animal was placed in the lighted section and the time until it moved to the dark section was measured. An electric stimulus (0.08 mA) was applied 5 s after the mouse entered the dark section. The test was repeated 24 h after the training session. The first training test lasted 180 s and the repeated test 300 s.

### 2.9. Histological Analysis

Morphological assessment of mouse brain tissue was carried out according to a published protocol [31]. In brief, the surgically isolated brains were fixed in 10% formalin solution and then incubated in sucrose solutions of increasing concentration (15% and 30%) for a total of 48–72 h. The samples were gradually filled with cryogel (Leica, Wetzlar, Germany) and cut into 10 µm coronal sections using a Leica CM1520 freezing sliding cryostat (Leica, Germany). The sections were stained with hematoxylin–eosin (PanReac AppliChem, Germany), and examined with a Zeiss Primo Star light microscope (Zeiss, Germany) with an integrated Axio CamMRc camera (Zeiss, Germany).

### 2.10. Statistical Analysis

Statistical analysis was performed using GraphPad Prism v.9.3.1.471 (San Diego, CA, USA). The results are presented as the mean ± standard error of the mean (SEM). The Shapiro–Wilk test was used for normal distribution analysis. Differences between two independent groups were assessed by the Mann–Whitney test. Epi-fluorescence imaging data were analyzed using two-way ANOVA. Differences between groups were considered significant if the corresponding *p*-value was less than 0.05.

## 3. Results

### 3.1. Creation of the Stable Fluorescent Murine Glioma GL261-Kat Cell Line

We created a novel, stable, monoclonal cell line of murine glioma expressing the far-red fluorescent protein, TurboFP635. The parental murine glioma, GL261, is a well-characterized cell line commonly used in experimental murine glioma models [34]. TurboFP635 has broad emission in the far-red spectral range, with a maximum at 635 nm and right shoulder up to 750 nm, which makes it suitable for deep whole-body imaging. Importantly, TurboFP635 fluorescence is several times brighter (due to high extinction and fluorescence quantum yield) than other proteins with similar spectral properties, including HcRed and mPlum, and it is highly photostable and stable against pH changes [35].

**Figure 2:**
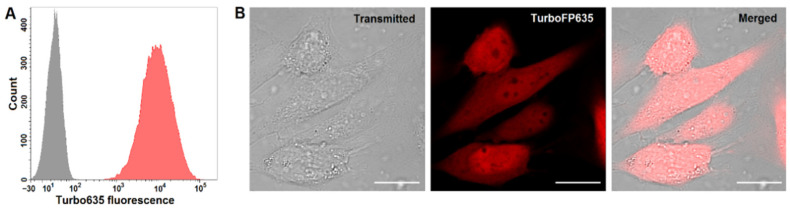
**Establishment and characterization of GL261-kat cell line with TurboFP635 expression.** (**A**) GL261-kat cells have red fluorescence about three orders of magnitude higher than the autofluorescence of the parental GL261. Shown are cell distributions of parental cell line (grey) and monoclonal GL261-kat (red), according to fluorescence intensity: λ_ex_ 651 nm; λ_em_ 610–630 nm. (**B**) The fluorescent protein is homogenously distributed in cell cytoplasm and nuclei. The confocal images of GL261-kat cells were obtained using an Axio Observer Z1 LSM-710 DUO NLO laser scanning microscope (Carl Zeiss, Germany). Shown are transmitted light image, fluorescent image (λ_ex_ 594 nm; λ_em_ 603–720 nm), and image with merged channels. Scale bars, 20 μm.

The GL261 cells were transfected by lipofection and primary selection was performed on an antibiotic-containing medium, with multiple rounds of optical sorting. Long-term repeated passaging allowed for the selection of individual cells with stable expression, presumably as a result of chromosomal integration of the fluorescent protein gene. As a result, we obtained monoclonal GL261-kat cells with red fluorescence of about three orders of magnitude higher than the autofluorescence of parental GL261 cells when analyzed by flow cytometry (Figure 2A). In GL261-kat cells, the fluorescent protein is homogenously distributed in the cytoplasm and nuclei (Figure 2B). The latter phenomenon proves the ability of Turbo635 to passively localize in the nuclei without the need for nuclear localization sequence and translocation-assisting proteins. Of note, such nuclear penetration is widely reported for small proteins with a molecular weight below 60 kDa, including those of GFP-family [13,36]. Of note, GL261-kat exhibits a morphology and growth patterns similar to the parental GL261 cell line. Therefore, a serious impact of the transfection procedure on cell metabolism is excluded. The stability of TurboFP635 expression is proven through more than 30 passages and also after cryopreservation. This stability enabled us to use the novel GL261-kat cell line in an orthotopic mouse glioma model.

### 3.2. Morphological Alterations of Brain Tissue in Orthotopic Mouse Glioma Model

Next, we assessed the features of glioma progression in the orthotopic mouse tumor model by using the glioma GL261-kat cells. To examine the ability of the new cell line to maintain fluorescence for a long time, we used allogenic BALB/c mice in order to decrease the intensity of tumor progression [29,30]. We intracranially injected the mouse brains with 2.5 × 10^4^ GL261-kat cells (GL25 group) or with 5 × 10^4^ cells (GL50 group). The control mice were intracranially injected with the appropriate volume of PBS.

The intensity of tumor growth was assessed over a five week period by analyzing the morphological changes in the brain tissue using lifetime in vivo imaging, as well as by histological analysis. We also quantified neurological deficit, general locomotor activity, and orienting-exploratory activity of the mice in the “Open Field” setup, and cognitive and learning abilities in the passive avoidance test.

**Figure 3:**
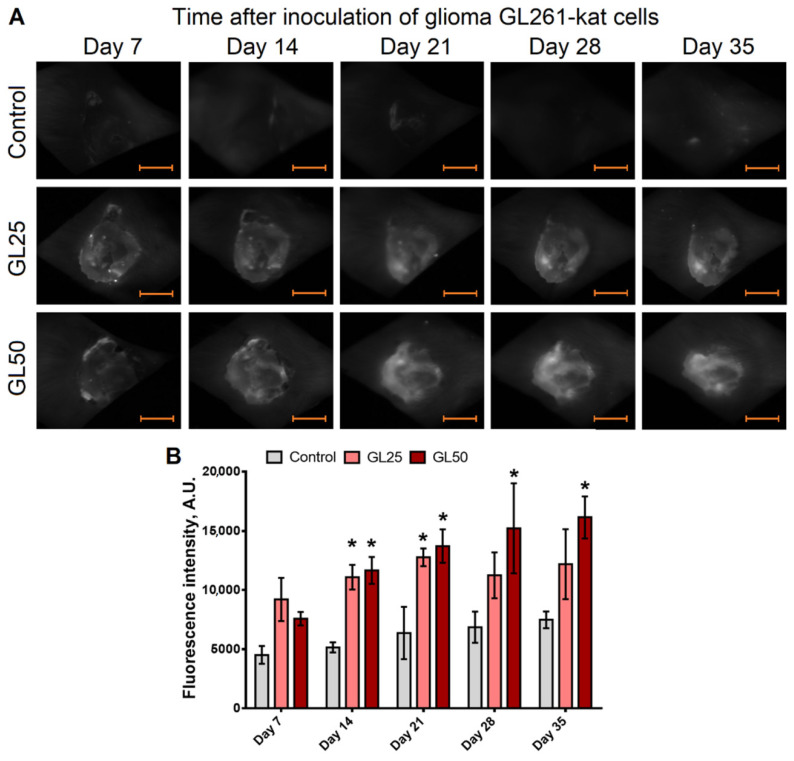
**Epi-fluorescence imaging assessment of mouse brains after inoculation of glioma GL261-kat cells.** (**A**) The glioma cells, 2.5 × 10^4^ cells (GL25 group) or 5 × 10^4^ cells (GL50 group) resuspended in PBS (2 µL), were injected 2.5 ± 0.5 mm below the dura mater. The control mice were injected intracranially with the appropriate volume of PBS. TurboFP635 was detected (λ_ex_ 590 nm; λ_em_ 625–675 nm) by using a whole-body imaging system (Deep Vision System, DVS-03, Russia). Scale bars, 5 mm. (**B**) The fluorescence intensity of glioma GL261-kat cells in mice enables us to confidently detect these cells starting on day 14 after inoculation, and the fluorescence signal intensity increases with tumor progression. The integral fluorescent signal in the GL50 group mice have a tendency to increase relative to the GL25 group on day 35 after inoculation. * *p* < 0.05 versus control, two-way ANOVA with Dunnett multiple comparisons test, *n* = 4.

Using in vivo epi-fluorescence imaging, we are able to confidently detect the fluorescence of glioma growing in mice in both the GL25 and GL50 groups (Figure 3).

On day 14 after inoculation of glioma GL261-kat cells, the glioma cells spread across the surface of the cerebral cortex in the sagittal plane (Figure 3). At that time, an increase in fluorescence is detected in 60% of the mice. Later, the fluorescence signal continues to increase with the glioma progression. On day 21 after inoculation, the formation of fluorescent glioma without clear boundaries is observed in the brain of all the experimental mice. The integral fluorescent signal in the GL50 group is noticeably higher than in the GL25 group, which is consistent with the amount of initially inoculated glioma cells.

**Figure 4:**
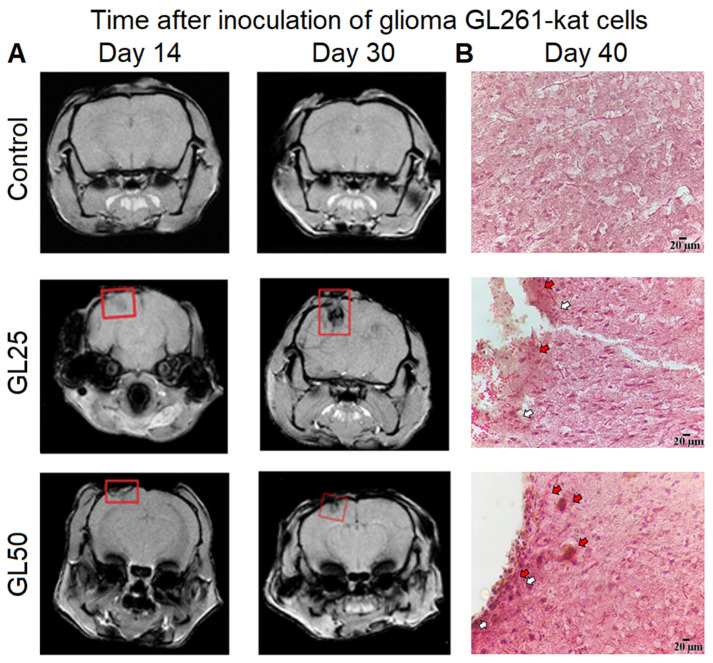
**Dynamics of morphological changes of mouse brain tissue after inoculation of glioma GL261-kat cells.** (**A**) Representative magnetic resonance images (MRI) of mouse brains on days 14 and 30 post-injection. The multi gradient echo multi slices (MRI MGEMS) were obtained by a high-field magnetic resonance tomograph, Agilent Technologies DD2-400 9.4 T (400 MHz) and the following parameters were assessed: TR = 1000 ms, TE = 1.49 ms, 6 echoes, FOV 20 mm × 20 mm, matrix 256 × 256, slice thickness 1 mm, 15 slices, 17 min and 4 sec scanning time. The red squares indicate the area of injection of glioma cells and tumor growth. MR tomograms reveal the presence of a heterogeneous structure in the right parietal lobe of mouse brains in both the GL25 (2.5 × 10^4^ cells) and GL50 (5 × 10^4^ cells) groups on day 14 after inoculation of glioma cells, and the volume of the glioma site tends to increase by day 30 in the post-injection period. (**B**) Histological samples of brain cortex of mice were obtained on day 40 after inoculation of glioma GL261-kat cells. Hematoxylin–eosin staining, magnification ×20. The brain tissue of the control group has a typical morphology without significant changes in the vascular system structure. In the GL25 group, approximately at the site of glioma cells injection, tumor cells with vague, small nuclei and atypical neuronal cells with an elongated nucleus and small necrotic sites (white arrows) are observed. Red arrows indicate the local points of infiltrative tumor growth. In the GL50 group, a dense accumulation of tumor cells of various shapes and sizes is observed at the edge of histological preparations. Tissue edema and active necrotic process can also be seen (white arrows). Scale bars, 20 µm.

In parallel, the dynamics of morphological changes in mouse brain tissue after inoculation of glioma GL261-kat cells were assessed using MRI (Figure 4A). MR tomograms obtained on day 14 after surgery reveal tinted areas in the right parietal lobe of the brains in both the GL25 and GL50 groups, indicating the presence of a heterogeneous structure (Figure 4A). On day 30 after inoculation, glioma without clear boundaries with healthy brain tissue is seen in 20% of mice in the GL25 group. The volume of the glioma site increases prominently (day 14: 11.3 ± 4.4 mm^3^, day 30: 38.5 ± 16.4 mm^3^). In comparison with the 14th day of the post-injection period, no pronounced morphological alterations of brain tissue are observed on day 30 after glioma cells inoculation in the GL50 group. However, the volume of the glioma tends to increase (day 14: 8.5 ± 0.8 mm^3^, day 30: 13.0 ± 4.3 mm^3^).

Histological analysis of the brain cortex was performed on day 40 after inoculation of glioma GL261-kat cells (Figure 4B). No significant morphological changes of brain tissue are observed in the control group (Figure 4B). Neurons with numerous processes are evenly arranged without clustering, and are predominantly oval or circular shape with centrally located nuclei occupying 60% to 90% of cell size; the cytoplasm is uniform, and the structure of the vascular system has a typical morphology. Analysis of histological preparations from the GL25 group reveals neuronal cells of various sizes with atypical, elongated nuclei (Figure 4B). At the site of GL261-kat cells injection, approximately 20% of the tumor cells have vague, small nuclei, and 15% of the neuronal cells had atypical, elongated nuclei. In 10 fields of view of the histological sample, the presence of 1–2 small necrotic brain tissue sites is also seen. In the GL50 group, a dense accumulation of numerous tumor cells of various shapes and sizes is observed at the edge of the histological preparations (Figure 4B). The nerve cells have indistinct borders and blurred nuclear outlines, indicating tissue edema and an active necrotic process. The brain cortex of mice in the GL50 group is also characterized by a large number of round and oval blood vessels with thin walls, which form vascular clusters. Dense clusters of tumor cells are located close to the vessels. The development of a vascular network in close interaction with atypical cells indicates an active tumor process.

### 3.3. Assessment of Behavioral and Cognitive Reactions of Mice in the Orthotopic Glioma GL261-Kat Model

**Figure 5:**
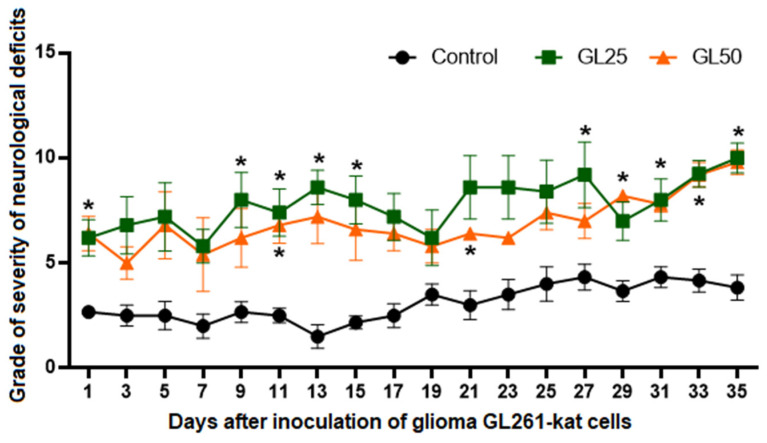
**Neurological status assessment of mice after inoculation of glioma GL261-kat cells.** The data demonstrate that inoculation of 2.5 × 10^4^ glioma cells (GL25) or 5 × 10^4^ cells (GL50) results in gradual development of neurological deficits, reaching scores of 10.7 ± 0.3 (GL25) and 9.8 ± 0.6 (GL50), respectively, by day 35 post-injection, which correspond to severe central neural system damage. No significant differences between the GL25 and GL50 groups are found throughout the observation period. * *p* < 0.05 versus controls, Mann–Whitney test.

The neurological status assessment reveals the gradual development of neurological deficits in both experimental groups (Figure 5). On day 35 after inoculation of glioma GL261-kat cells, the mean scores of neurological deficits in the GL25 and GL50 groups are 10.7 ± 0.3 and 9.8 ± 0.6, respectively, which correspond to severe central neural system damage. However, no significant differences between the two experimental groups are found throughout the observation period.

The development of neurological deficits is accompanied by changes in general locomotor and orienting-exploratory activity of the experimental mice (Table 1). Analysis of behavioral reactions in the open field test of mice from the GL25 group reveals a significant increase in the time spent in the arena center, which indicates a decrease in the level of fear and anxiety. Pronounced changes in the orienting-exploratory activity in the GL25 group are also observed. The alterations are characterized by an absence of upright postures and a significant decrease in the number of peeks into the arena’s holes, indicating impaired mink reflex repeatability. The mice in the GL50 group also show less pronounced motor and orientation-exploratory activity compared to the control group (Table 1). There is a decrease in the number of peeks in holes and squares passed in the periphery of the arena accompanied by a tendency to spend more time in the arena center.

In addition, the effect of inoculation of glioma GL261-kat cells on cognitive functions and learning ability was analyzed using the passive avoidance test (Table 2). During the training session, the mice enter the dark chamber section and tend to leave the brightly lit space, following the mink reflex. However, in the GL50 group, the latent time of movement to the dark section is significantly longer than in the control group. A similar tendency is shown by the GL25 group. These observations are correlated with the open field data, and indicate a decrease in motivation to avoid bright light and open space.

The latent period of movement to the dark section in the retest significantly increases in the control and experimental groups relative to the training session. Thus, after inoculation of glioma GL261-kat cells, the mice preserve learning ability and memorial trace in response to an electrical stimulus. No significant differences relative to the control group are seen.

## 4. Discussion

In this study, a novel fluorescence glioma GL261-kat cell line stably expressing a far-red fluorescent protein TurboFP635 (Katushka) was generated and characterized. Using an orthotopic mouse glioma model and epi-fluorescence imaging, we demonstrate confident detection of the fluorescence of glioma GL261-kat cells in mice, and an increase in the fluorescence signal during glioma progression accompanied by gradual development of neurological deficits and behavioral alterations in mice.

The murine GL261 cell line is widely used in experimental mouse models to study glioma biology. Ausman and colleagues were the first to report the generation of GL261 by intracranial injection of the alkylating agent (i.e., 3-methylcholantrene) into C57BL/6 mice, and their maintenance in syngeneic mice using a series of intracranial and subcutaneous transplantations of tumor tissue [37]. The efforts of several research groups led to the creation, from the GL261 tumor, of a stable cell line, which was subsequently characterized in detail [34]. The glioma GL261 cell line has several advantages, such as a high aggressiveness accompanied by an easy adaptation to in vitro fast growth without contact inhibition. Moreover, the p53 gene mutation associated with elevated p53 expression in glioma GL261 cells brings this line closer in biological characteristics to human gliomas. Unlike the rat glioma C6 cell line, murine glioma GL261 cells do not spontaneously regress [34,38]. Moreover, GL261 cells are easy to transfect or virally transduce, which opens possibilities for the development of novel genetically modified fluorescent cell lines for experimental imaging of tumor growth in vitro and in vivo [39,40]. GL261 cells are moderately immunogenic and suitable for orthotopic implantation in syngeneic and immunocompetent animals to study tumor immunology [39,41]. For instance, glioma GL261 cells were used to study the mechanisms of immunogenicity of cell death in response to various anti-cancer therapeutic agents, including to chemotherapeutics and photodynamic therapy [42,43,44,45].

Glioma GL261 cells in the syngeneic orthotopic mouse model are characterized by rapid tumor growth accompanied by the development of severe neurological deficits and aberrations in behavioral reactions [46]. The tumor size and local tumor tissue extension can be monitored well by MRI from the second week after inoculation of glioma cells [47]. The relatively novel approaches for intravital assessment of glioma progression are based on optical-based techniques, combined with genetic labeling of glioma cells with luciferase [40,41] or fluorescent proteins. Due to the high-light-absorption in the visible spectral range, the green fluorescent proteins used in the initial studies were more applicable for ex vivo analysis [48]. Later, the creation of far-red emitting proteins gave rise to more relevant in vivo follow-ups of tumor progression and invasion [49,50]. While the optical penetration depth for green light in the brain tissue of both humans and rodents does not exceed 0.5 mm in blue/green spectral region, it increases up to 1.0–1.5 mm for red light [51,52]. It means that 2–5% on initial light flow penetrates at depths of about 3–4 mm, which is of interest for small animal glioma models. Despite the attractiveness of the approach, there are few available red-emitting GL261-derived cells lines, such as cell lines expressing DsRed2 (λem 587 nm) [50] and mCherry (λem 610 nm) [53]. Moreover, it should be noted that, in most cases, the available GL261-derivative cell lines have not been tested for the whole-body imaging, and have been mostly used for ex vivo morphometric studies [53]. The only found study with an attempt to quantitate the in vivo GL261 glioma progression was performed using the Kusabira Orange (λem 561 nm), the protein with the optical properties far from ideal for deep whole-body imaging [49]. To create the GL261-kat cell line, we chose the fluorescent protein TurboFP635, which has an optimal combination of far-red emission and high quantum yield, providing excellent brightness supported by high photostability. Notably, the comparative experimental analysis of the TurboFP635 protein, also known as Katushka, demonstrated its beneficial properties compared to other red fluorescent proteins such as mNeptune, mCardinal, and eqFP650 [54].

Importantly, because glioma cells are highly aggressive, the overall survival rate of mice does usually not exceeded three weeks [46,47]. Allogeneic models are less common, but are also used to study glioma biology [55,56,57]. In the current study, the use of an orthotopic glioma model in allogenic BALB/c mice made it possible to decrease tumor aggressiveness, and thereby prolong mouse survival, which opens the possibility to evaluate the ability of preserving the fluorescence of the glioma GL261-kat cells in the long-term period. We show that glioma GL261-kat cells can be used to accurately assess the brain tumor progression in vivo. The fluorescence of glioma GL261-kat cells in the mice is confidently detected from day 14 after inoculation of glioma GL261-kat cells, with further increases in the fluorescence signal during tumor progresses. The integral fluorescent signal is consistent with the amount of inoculated glioma GL261-kat cells. Furthermore, active tumor progression is confirmed by histological analysis. At the same time, the glioma growth is accompanied by the gradual development of neurological deficits and behavioral alterations in BALB/c mice. Thus, our data demonstrate the significance and feasibility of using the novel, fluorescently labeled glioma GL261-kat cell line as a model to study glioma biology in orthotopic mouse models and to analyze the initiation and invasion of glioma, and the possibility of non-invasive lifetime tracking of glioma cells, which can be especially useful when assessing the responses to anti-cancer therapy.

## 5. Conclusions

A new fluorescent glioma GL261-kat cell line stably expressing the far-red fluorescent protein, TurboFP635 (Katushka), is generated and characterized. Using the orthotopic mouse glioma model, we show that the GL261-Kat cell line can be employed as a useful tool to study glioma biology, and to accurately and non-invasively monitor the impact of tumor growth in the brain tissue in orthotopic mouse models.

## Figures and Tables

**Table 1:** Parameters of behavioral reactions of mice in the open field test on day 32 after inoculation of glioma GL261-kat cells. (**A**) Parameters of locomotor activity; (**B**) Emotional status characteristics.

**(A)**					
**Mouse Group**	**Number of Squares Passed in the Arena**		**Time in the Arena Center, s**	**Number of Upright Postures**	**Integral Fluorescence Intensity of Glioma GL261-Kat Cells in the Brain Tissue, A.U.** **(Day 35 after Inoculation)**
	**Periphery**	**Center**			
Control	40.2 ± 12.2	13.4 ± 4.4	100.8 ± 33.7	1.2 ± 0.5	7480 ± 709
GL25	16.75 ± 11.6	21 ± 7.8	245.5 ± 32.5 *	0	12189 ± 2954
GL50	11 ± 6.2 *	14 ± 6.5	233.8 ± 49.4	0.2 ± 0.1	16127 ± 1770 #
(**B**)					
**Mouse Group**	**Grooming Time, s**		**Number of Peeks in Holes**	**Acts of Urination**	**Acts of Defecation**
Control	3.4 ± 1.5		20.6 ± 5.9	0.6 ± 0.3	1.8 ± 0.9
GL25	1.3 ± 1.4		8.5 ± 2.2 *	0.3 ± 0.1	1.3 ± 0.7
GL50	3.8 ± 1.1		2.6 ± 1.6 *	0.4 ± 0.3	3.2 ± 1.4

* *p* < 0.05 versus control, Mann–Whitney test. # *p* < 0.05 versus control, two-way ANOVA with Dunnett multiple comparisons test.

**Table 2:** The reproducibility of the conditioned passive avoidance reflex in mice in the remote period after inoculation of glioma GL261-kat cells.

Experimental Group	Latent Period of Movement to the Dark Chamber Section, s		Integral Fluorescence Intensity of Glioma GL261-Kat Cells in the Brain Tissue, A.U. (Day 35 after Inoculation)
	Training Session	Retesting	
Control	27.2 ± 7.5	279.8 ± 22.1	7480 ± 709
GL25	54.0 ± 17.7	203.9 ± 67.9	12,189 ± 2954
GL50	78.5 ± 13.8 *	220.7 ± 54.4	16,127 ± 1770 #

* *p* < 0.05 versus control, Mann–Whitney test. # *p* < 0.05 versus control, two-way ANOVA with Dunnett multiple comparisons test.

## Data Availability

The data used to support the findings of this study are available from the corresponding author upon request.
